# Multimodal neuromonitoring in the nordic countries: experiences and attitudes – a multi-institutional survey

**DOI:** 10.1007/s00701-025-06479-7

**Published:** 2025-03-12

**Authors:** Anna Søgaard Magnussen, Markus Harboe Olsen, Anders Rosendal Korshøj, Tiit Mathiesen, Axel Forsse, Carsten Reidies Bjarkam, On behalf of the Danish Neurointensive Care Monitoring Consortium

**Affiliations:** 1 Department of Neurosurgery, The Neuroscience Centre, Copenhagen University Hospital – Rigshospitalet, Inge Lehmanns Vej 8, Copenhagen, 2100 Denmark; 2 Department of Neuroanesthesiology, The Neuroscience Centre, Copenhagen University Hospital – Rigshospitalet, Copenhagen, Denmark; 3 Department of Neurosurgery, Aarhus University Hospital, Aarhus, Denmark; 4 Department of Clinical Medicine, Aarhus University, Aarhus, Denmark; 5 Department of Clinical Medicine, University of Copenhagen, Copenhagen, Denmark; 6 Department of Clinical Neuroscience, Karolinska Institute, Stockholm, Sweden; 7 Department of Neurosurgery, Aalborg University Hospital, Aalborg, Denmark

**Keywords:** Multimodal neuromonitoring, Neurointensive care, Traumatic brain injury, Subarachnoid hemorrhage, Acute brain injury

## Abstract

**Background:**

Multimodal neuromonitoring (MMM) aids early detection of secondary brain injury in neurointensive care and facilitates research in pathophysiologic mechanisms of the injured brain. Invasive ICP monitoring has been the gold standard for decades, however additional methods exist (aMMM). It was hypothesized that local practices regarding aMMM vary considerably and that inter-and intracenter consensus is low. The survey aimed to investigate this hypothesis including the knowledge, attitudes towards, and use of aMMM in the neurointensive care setting in the Nordic countries.

**Method:**

The survey was distributed amongst 54 neurosurgical trainees at a Nordic neurosurgery training course and supplemented with 16 center-appointed neuromonitoring experts representing 16 of the 19 neurosurgical centers in the Nordic countries (Norway, Sweden, Denmark, and Finland).

**Results:**

The response rate was 100% amongst the training course attendents, as well as the center-appointed experts with a total of 70 respondents. The experts covered 16/19 Nordic centers. In-center disagreement was high concerning the use of aMMM methods. In patients with traumatic brain injury, subarachnoid hemorrhage, or other acute brain injuries 50% of the appointed experts stated transcranial Doppler ultrasound (TCD) to be used in most cases in their ICU, and an additional 25% for selected cases. Most appointed experts agreed on electroencephalography (EEG) for selected cases 63%, but only 19% for most cases. Routine use of Invasive brain tissue oxygenation (PbtO_2_) was stated by 25–63% and cerebral microdialysis (CMD) by 19–38%. The main perceived concerns with aMMM methods were the usefulness for outcome-changing interventions (43%) and financial issues (19%). Most respondents (67%) believed automated combined analysis of aMMM to be a likely future scenario.

**Conclusion:**

There was a remarkable variation in the reported use of aMMM among Nordic neurosurgical centers, indicating an extensive lack of consensus on need and utility. Surprisingly routine use of TCD was stated by 75%, presumably for routine monitoring of SAH patients, whereas CMD was mostly considered a research tool. Interestingly, junior staff and appointed experts disagreed on intended local routines, indicating that application of aMMM was more governed organically and by case than on explicit guidelines or that uniform management was not prioritized.

## Introduction

When the brain is injured, a myriad of pathophysiological mechanisms ensues. Through edema, hypoxia, perturbed energy metabolism, inflammation, and compromised cerebrospinal fluid dynamics secondary injury may occur [43]. Multimodal neuromonitoring (MMM) is the combined implementation of various cerebral monitoring techniques in the treatment of neurocritical patients. These techniques allow the detection of imbalances in the brain, early recognition of secondary brain injury, and enable personalized treatment [24, 48, 51].

Intracranial pressure (ICP) monitoring was the first continuous monitoring technique to gain wide acceptance internationally in the 1980s and has been the gold standard for monitoring severe TBI and SAH. However, it is unable to early detect other secondary threats including hypoxia, epileptiform activity, and disturbed metabolic function [32, 46]. Several additional neuromonitoring methods (aMMM) have been developed and combined for clinical and research-related use. Objects of monitoring include electrophysiologic activity measured via non-invasive electroencephalogram (EEG), cerebral tissue oxygenation measured by invasive techniques; brain tissue oxygen tension (PbtO_2_) and jugular bulb oxygen saturation (SjvO_2_) or by non-invasive; near-infrared spectroscopy (NIRS), cerebral metabolism measured invasively by cerebral microdialysis (CMD), vasospasms non-invasively measured via transcranial Doppler ultrasonography (TCD), and cerebral blood flow (CBF) invasively measured via thermal diffusion flowmetry (TDF).

MMM is recognized as a cornerstone of modern neurocritical care, and its use is recommended by international forums and societies [8, 23], yet variations in monitoring indications and interpretation occur. The Nordic countries are, in an international context, arguably homogenous in health care standards and health care organization with extensive multilateral collaboration. However, there have been no prior investigations of the extent of use and attitudes towards aMMM methods in this region, resulting in doubts of current standard of care and research as well as inter-center comparability. Furthermore, an exploration of knowledge and opinions toward risks, cost–benefit and prioritization among future neurosurgeons may be important for optimization of targeted patient care. It was the hypothesis of the Danish Neurointensive Care Monitoring Consortium that local practices regarding aMMM vary considerably and that inter- and intra-center consensus is low. This survey aimed to describe the extent of aMMM use, clinically and in research. Additionally, opinions towards risks, cost–benefit, prioritization, and future development of the concerned techniques are explored.

## Methods

In this multi-institutional survey, a questionnaire regarding knowledge of- and attitudes toward the use of aMMM methods in the neurointensive care setting was distributed among neurosurgical trainees and senior physicians (appointed experts) employed in Nordic neurosurgical departments and in the neurointensive care unit (NICU). The respondents were asked to complete the questionnaire anonymously and individually. Neurosurgical trainees were approached in March 2022, during the Beitostølen Scandinavian Neurosurgery Course, which for many centers is a mandatory module to achieve a specialist degree. From November to December 2023 each of the 16 Neurosurgical centers was contacted by mail and asked to forward the questionnaire to the doctor responsible for each center’s use of aMMM (appointed experts), to obtain a benchmark and investigate in-center agreement. Reminders to complete the survey were sent out during the data collection period to minimize nonresponse.

The questionnaire contained 20 topic-specific multiple-choice questions with a variety of 2–5 possible answers, which can be divided into three main themes:

- *Theme 1*: Background: home country and center, level of training, and seniority.
- *Theme 2*: Home center policy regarding the use of; CMD, PbtO_2_ individually and combined, NIRS, TCD, cEEG, SjvO_2_, TDF, and radiological estimation of CBF.
- *Theme 3*: Opinions towards the interest, knowledge, implementation, main problems, and further perspectives.

This survey does not contain patient data or clinical studies with human participants or animals performed by any of the authors, thus human ethics and consent to participate were not applicable.

Data was entered and analyzed using descriptive statistics in Excel and SPSS. Data regarding the use of aMMM were grouped in “clinical use”, “research use” and “not in use” before calculating disagreement using a weighted linear kappa score in SPSS v29. The kappa score was interpreted as low (subdivided into no, minimal, and weak) or high (subdivided into moderate, strong, and almost perfect) agreement as suggested by McHugh in 2012 [34]. Missing data were presented for each question (Electronic Supplementary Material).

## Results

The survey had a 100% response rate with 70 participants. Fifty-four were trainees and 16 were appointed experts from 16 of the 19 neurosurgical centers in the Nordic countries. Two experts and one trainee were Neurointensivitst, whereas the rest were neurosurgeons or neurosurgical trainees. The ratio of trainees to appointed experts in each country was similar, with a variation of 3–3.7 trainees per appointed expert between the countries (Denmark 3:10, Finland 3:10, Norway 4:12, and Sweden 6:22). Missing answers occurred with a frequency of 0–9% per question (Electronic Supplementary Material).

**Fig. 1:**
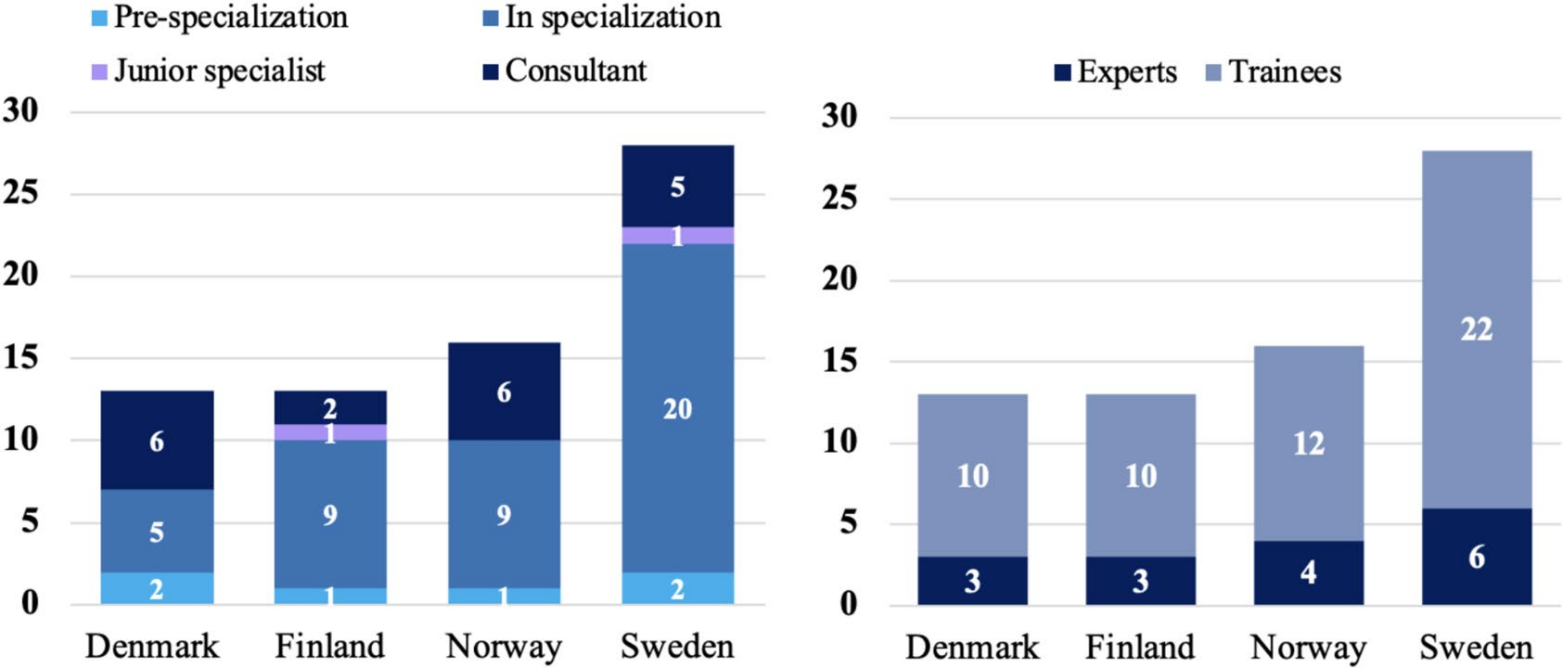
Descriptive data of respondents | (**A**) A bar chart showing the seniority of the 70 respondents. **B** A bar chart showing the number of respondents per country

Respondents in specialization and pre-specialization were course participants, whereas the consultants and junior specialists were the instructors of the course and the appointed experts from each center (Fig. 1). According to the appointed experts, 12 (75%) stated that NICU rounds were mainly handled cooperatively by both neurosurgeons and neurointensivists.

### Use of multimodal neuromonitoring

**Fig. 2:**
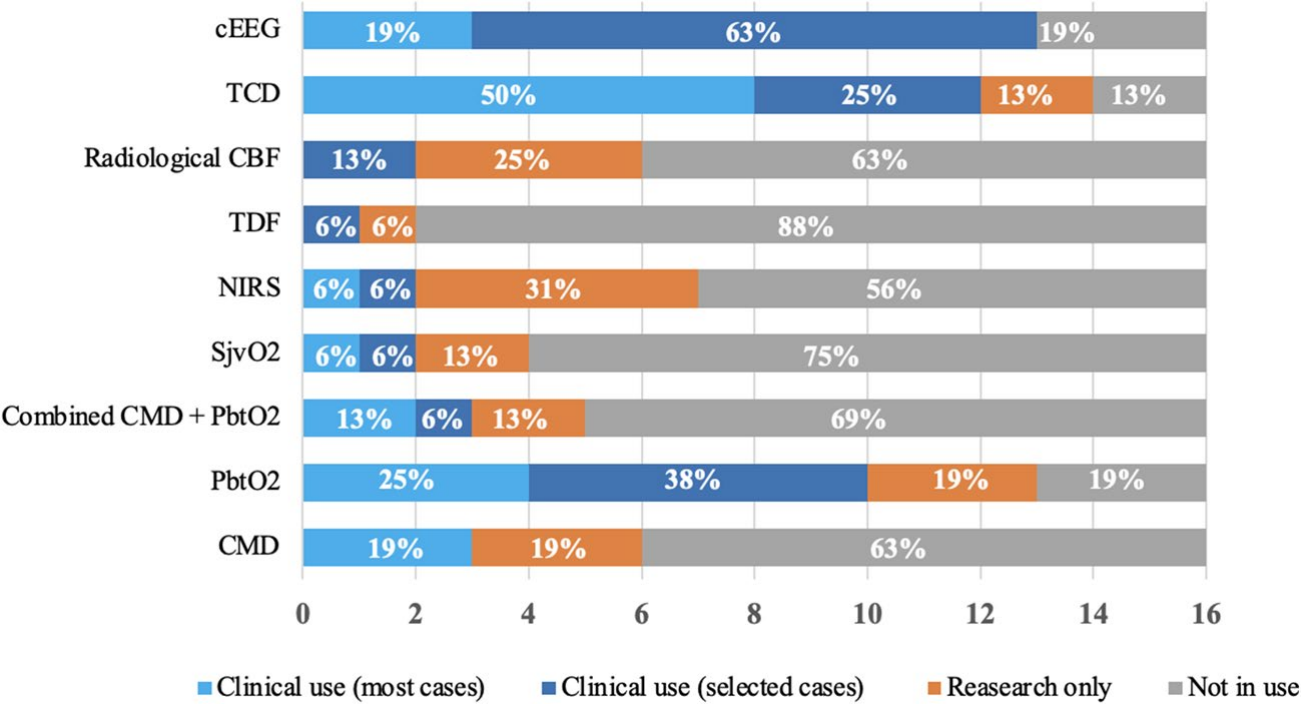
Use of multimodal neuromonitoring in the Nordic countries – appointed experts’ answers. Stacked bar chart showing the distribution between research use, clinical use in most or selected cases, and no use in 16 Nordic neurosurgical centers reported by the appointed experts from each center. *Abbreviations: CBF, cerebral blood flow; cEEG, continuous electroencephalogram; CMD, cerebral microdialysis; NIRS, near-infrared spectroscopy; PbtO2, brain tissue oxygenation; SjvO2, Jugular bulb oxygen saturation; TCD, transcranial Doppler; TDF, thermal diffusion flowmetry*

**Fig. 3:**
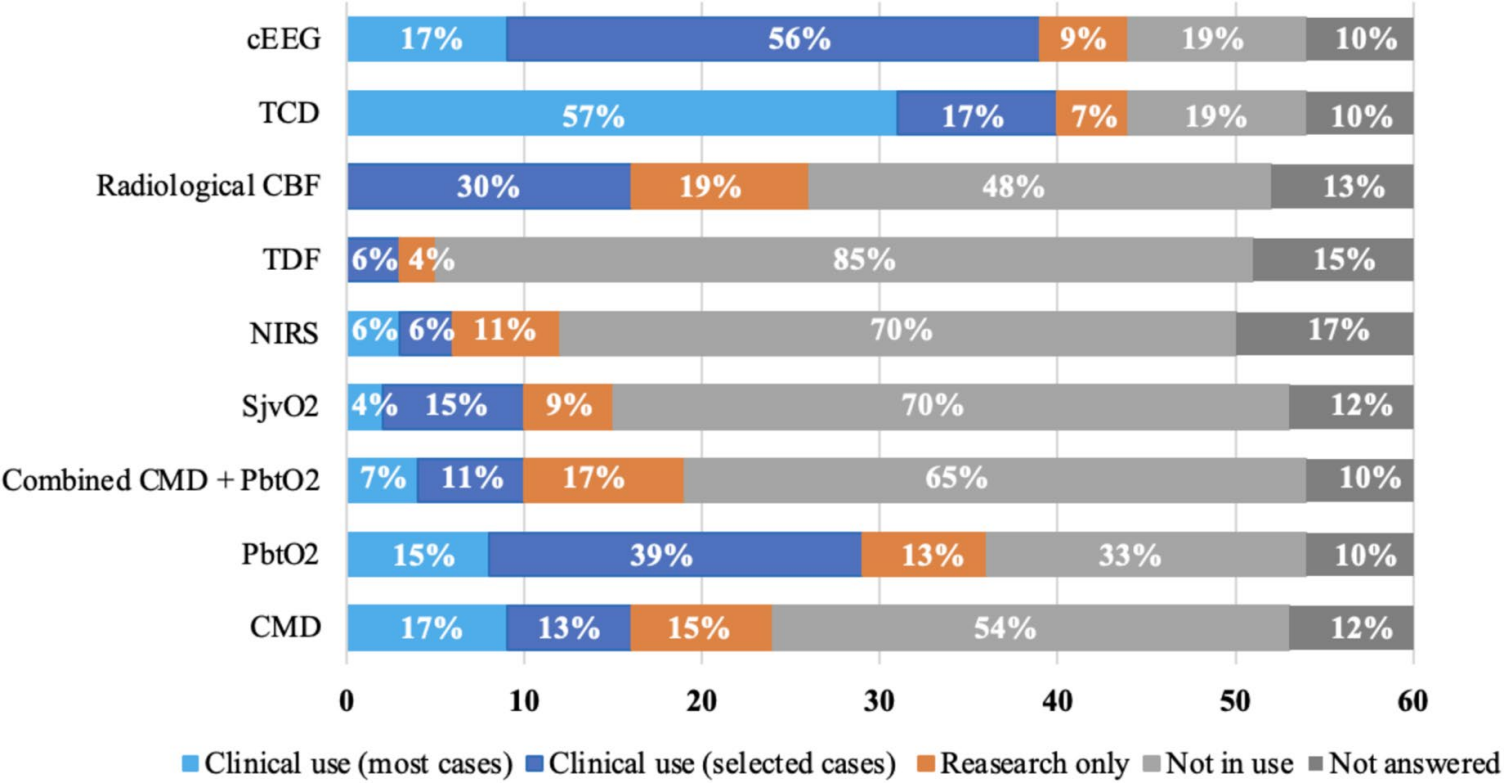
Use of multimodal neuromonitoring in the Nordic countries – trainees’ answers. Stacked bar chart showing the distribution between research use, clinical use in most or selected cases, and no use in 16 Nordic neurosurgical centers reported by the trainees. Numbers may not add up to 100% (70 responders) due to missing answers. *Abbreviations: CBF, cerebral blood flow; cEEG, continuous electroencephalogram; CMD, cerebral microdialysis; NIRS, near-infrared spectroscopy; PbtO2, brain tissue oxygenation; SjvO2, Jugular bulb oxygen saturation; TCD, transcranial Doppler; TDF, thermal diffusion flowmetry*

**Fig. 4:**
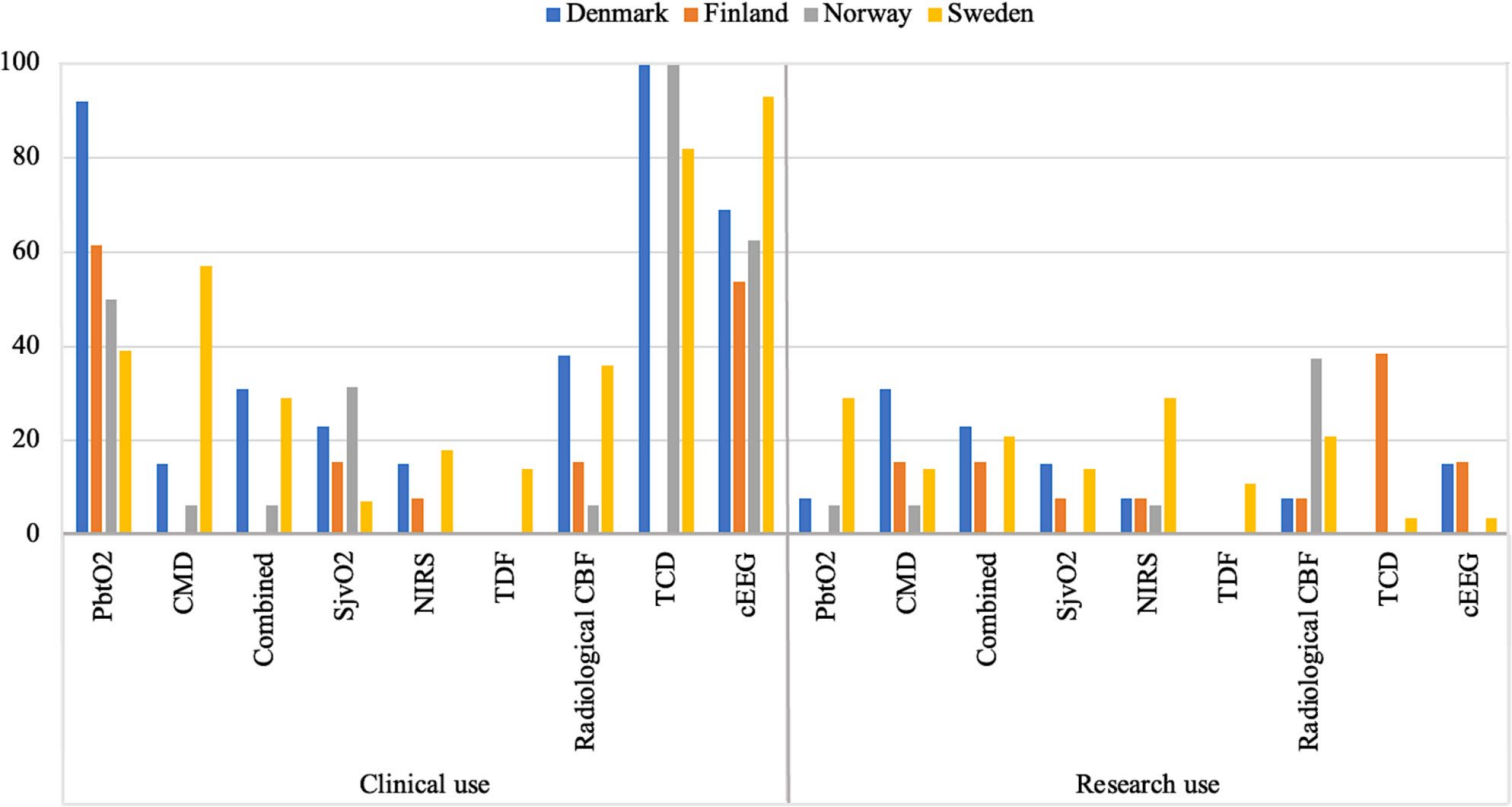
Use of multimodal neuromonitoring per country – appointed experts and trainees. Bar chart showing the distribution between research use, clinical use, and no use in 4 Nordic countries. Numbers may not add up to 100% due to missing answers. The use is shown in the percentage of responders per country. *Abbreviations: CBF, cerebral blood flow; cEEG, continuous electroencephalogram; CMD, cerebral microdialysis; NIRS, near-infrared spectroscopy; PbtO2, brain tissue oxygenation; SjvO2, Jugular bulb oxygen saturation; TCD, transcranial Doppler; TDF, thermal diffusion flowmetry*

According to the center-appointed experts, clinical use of certain neuromonitoring modalities outweighed research-driven use by far. Major differences were seen regarding the use of EEG (clinical use 81% vs research 0%), TCD (clinical use 75% vs research 13%), and PbtO_2_ (clinical use 62% vs research 40%), which were the three most clinically used methods. The remaining modalities were used in less than 19% of the centers. Even though EEG was available in most centers, frequent use (in most NICU cases) was only stated by 19% of appointed experts **(**Fig. 2**).** The research use was reported to be lower by the trainees in most modalities, except for EEG and the combined use of CMD and PbtO_2_ **(**Fig. 3**).** There was a clear difference when calculating the use of modalities per country. Sweden was the only country using all modalities in clinical routine, whereas Finland was only using five out of nine modalities **(**Fig. 4**).**

### Level of agreement concerning multimodal neuromonitoring

The in-center level of trainee-expert agreement was low among 61%, and only five trainees (9%) had a strong to almost perfect level of agreement with their respective experts **(**Table 1**).** Between experts from different centers within the same country, the level of agreement was low in all countries. (Electronic Supplementary Material).

**Table 1:** In-center level of agreement between experts and trainees concerning the use of aMMM

Level of agreement (Value of K)	%Data reliability	Trainees (Number)	Mean Kappa	Mean 95% CI
None (0–0.2)	0–4%	9.3% (5)	0.06	−0.23–0.48
Minimal (0.21–0.39)	4–15%	22.2% (12)	0.31	−0.15–0.71
Weak (0.40–0.59)	15–35%	29.6% (16)	0.49	0.08–0.82
Moderate (0.60–0.79)	35–63%	29.6% (16)	0.68	0.36–0.99
Strong (0.80–0.90)	64–81%	7.4% (4)	0.87	0.67–1.06
Almost perfect (> 0.9)	82–100%	1.9% (1)	0.93	1.00–2.00

Weighted Kappa score calculated between the appointed expert from each center and each of the respective trainees. The level of agreement was categorized based on the Kappa score

### Interest, knowledge, and attitudes towards CMD and PbtO_2_

Interest in aMMM was perceived as equal among concerned neurospecialists. While 71% of respondents claimed knowledge of CMD mechanisms, only 41% were confident in the interpretation and use of PbtO_2_ and CMD. In the appointed expert group, a higher proportion claimed knowledge, experience from research activities, and confidence in the interpretation and use of CMD and PbtO_2_ compared to the trainees. Furthermore, appointed experts reported a higher lack of interest than trainees (Table 2).

**Table 2:** Answers from appointed experts and trainees, Sect. 3 in the questionnaire

	Appointed experts	Trainees	All
Interest from neurosurgeons	*N*=16	*N*=54	*N*=70
Very true	6%	11%	10%
True	63%	52%	54%
N/A	0%	24%	19%
Untrue	31%	9%	14%
Very untrue	0%	4%	3%
Interest from neurointensivists	*N*=16	*N*=54	*N*=70
Very true	25%	17%	19%
True	31%	50%	46%
Untrue	13%	7%	9%
Very untrue	6%	4%	4%
None of the above	25%	22%	23%
Aware of CMD and PbtO2 mechanisms	*N*=16	*N*=54	*N*=70
Yes	100%	63%	71%
No	0%	35%	27%
Unsure	0%	2%	1%
Confident in use and interpretation of CMD and PbtO_2_	*N*=16	*N*=54	*N*=70
Yes	56%	37%	41%
No	44%	63%	59%
Used CMD and/or PbtO_2_ for research purposes	*N*=16	*N*=54	*N*=70
Yes	75%	24%	36%
No	25%	76%	64%
Currently using CMB or PbtO_2_ for research	*N*=16	*N*=54	*N*=70
Yes	50%	4%	14%
No	50%	96%	86%
Believe the implementation of CMB and/or PbtO_2_ is	*N*=16	*N*=48	*N*=64
A sound clinical practice allowing early detection of secondary brain injury	69%	46%	51%
A practical and safe way to study pathology and effects of intervention	19%	37%	33%
An unnecessary surgical risk	13%	6%	7%
The main issues with extended neuromonitoring is	*N*=16	*N*=49	*N*=65
Financial costs	13%	20%	19%
Time consumption	13%	13%	13%
Surgical risk	0%	15%	11%
Usefulness of measurements concerning interventions	56%	39%	43%
Usefulness of measurements concerning prognostics	19%	4%	7%
Believe automated data-analysis is going to be standard in the future	*N*=16	*N*=53	*N*=69
Yes	69%	67%	67%
No	0%	4%	3%
Maybe	31%N	28%	29%
Experience with the use of AI in neurointensive care	*N*=16	*N*=53	* N*=69
Yes	31%	15%	19%
No	69%	83%	80%

Numbers may not add up to 100% due to missing answers

*CMD*; cerebral microdialysi, *PbtO2*; brain tissue oxygenation, *AI*; artificial intelligence

Half of the respondents (51%) believed the implementation of CMD and/or PbtO_2_ to be a sound clinical practice. The appointed experts were more likely (69%) to agree with this than trainees (46%). The main concerns regarding aMMM were usefulness regarding outcome-changing interventions (43%), financial issues (19%), time consumption (13%), surgical risk (11%), and usefulness concerning prognostics (7%) (Table 2).

When asked for general comments, lack of funding, evidence, availability, and time were repeatedly mentioned by the appointed experts. Technical problems were specifically mentioned regarding CMD and PbtO_2_ monitoring. Other problems mentioned were the handling skills, interest, and awareness of the individual surgeon.

## Discussion

This survey aimed to determine the current use of aMMM in Nordic countries and explore the knowledge, opinions, and experiences towards the different aMMM modalities among experienced medical staff and the future generation of neurosurgeons. The most used aMMM modalities were EEG, TCD, and PbtO_2,_ and financial resources as well as the level of evidence for guided interventions were perceived as the main issues. In-center disagreement at the neurosurgical trainee-level was high.

### Current use of multimodal monitoring in the Nordic countries

#### EEG

EEG is commonly used to detect seizures and has been recommended in TBI patients by consensus conferences [8, 30], as post-traumatic subclinical seizures could be missed from a clinical assessment alone [5, 28]. Using scalp EEG to detect SD is mentioned as uncommon outside research settings in the Brain Trauma Foundation (BTF) guidelines [3]. In high-grade aSAH patients, monitoring of cEEG can be useful to predict DCI according to guidelines from the American Stroke Association (ASA) [22]. However, insufficient evidence exists to determine the long-term outcome effect of treatments based on cEEG monitoring [22]. Limitations include disturbance in EEG signals and the need for neurophysiologists to interpret the data [4]. EEG was stated to be used in most centers by the appointed experts (81%), though most commonly in selected cases (63%). This reflects a high accessibility to the method. In the questionnaire, continuous EEG was specifically queried, but considering the results, it was obvious that most respondents did not interpret this as different from regular EEG. In a Survey from the United States in 2024, cEEG was likewise found to be the most available non-invasive modality, however, MMM was the only standard of care according to 28% of the respondents [27].

#### TCD

Transcranial Doppler ultrasound measures flow velocity in the medial cerebral artery to detect vasospasms and may, under certain conditions, reflect changes in ICP [32, 42]. According to ASA guidelines, TCD is reasonable to detect vasospasms and predict DCI in aSAH patients [22]. However, TCD is not recommended in BTF guidelines due to a lack of high-quality evidence [3]. TCD was stated to be used in 12 (75%) of the Nordic centers. Interestingly, none of the appointed experts in Finland stated clinical routine use of this modality. When asked to elaborate, they answered it was due to previous experience with high interobserver variability and it being too time-consuming. Furthermore TCD can be limited by patient anatomy and affected by physiological measures [22].

#### PbtO_2_

Cerebral hypoxia can be present without increased ICP and has been associated with poor outcome [33, 36]. PbtO_2_ probes inserted in the brain parenchyma monitor local oxygenation, which enables treatment resulting in reduced brain hypoxia [2, 38]. According to ASA guidelines PbtO_2_ may be considered in aSAH patients, whereas PbtO_2_is is strongly supported as the second-most important monitoring variable after ICP, by the Seattle International Severe TBI Consensus Conference in 2019 [8, 22]. However, in a RCT study from 2023, the use of ICP and PbtO_2_ did not reduce poor outcome at six months compared to ICP monitoring only, yet it might be useful in subgroups of patients with severe traumatic brain injury [40]. Two multicenter randomized controlled trials studying PbtO_2_ monitoring in severe TBI are currently ongoing [31]. PbtO_2_ monitoring was the third most used method in the Nordic centers (63%). An essential limitation is probe location, as measurements are highly local [18].

#### Less commonly used neuromonitoring modalities

Cerebral oxygenation can also be measured invasively by a catheter in the jugular bulb (SjvO_2_) or non-invasively via spectrometry (NIRS) [37]. NIRS provides local information on tissue respiration from the hemoglobin concentration in the vessels [18], whereas SjvO_2_ provides global information [52].

BTF guidelines conclude a lack of high-quality data concerning SjvO_2_ [3]. A systematic review from 2014 found no evidence of improved outcome using NIRS in adult neurocritical patients [37]. Furthermore, low sensitivity to ischemia and high signal interference have been shown [13, 18, 19, 50]. SjvO_2_ and NIRS were used clinically in 2 centers (13%). The low use of SjvO_2_ could be due to less accuracy of SjvO_2_ compared to PbtO_2_, which has been shown in multiple studies [15, 17, 20, 25]. One reason for this is poor signal quality caused by e.g., inaccurate placement, clot formation, and inadequate calibration [10, 29, 41].

Detection of metabolic function via microdialysis was developed in 1974, and more than 16.500 papers have been published on the technique since [1]. Local metabolic changes occur preceding imaging detection of silent infarctions [21]. Monitoring the lactate-to-pyruvate ratio (LPR) via CMD enables detection of ischemia and mitochondrial dysfunction [47]. Interventions based on CMD changes could potentially prevent ischemia and infarction [21, 49]. However, there is no high-quality evidence showing improved outcome in CMD-monitored aSAH or TBI patients, and CMD is mentioned as uncommon outside research settings [3]. In this Survey CMD was used clinically in 3 (19%) neurosurgical centers. The clinical use in Europe and the US in 2023 was likewise low with about 30 centers [47]. Reasons for the low clinical use of CMD (19%) compared to PbtO2 (63%) may include handling obstacles, time delay, uncertainty regarding the interpretation of data and when and how to intervene [47]. Furthermore, multiple parameters and ratios can be analyzed from CMD, which makes it more difficult to interpret compared to PbtO_2_.

CBF can be monitored by radiological estimation or TDF. The risk of infarction depends on the degree and duration of decreased CBF, which has an essential effect on the outcome [11]. In TDF an invasive probe with a neutral and heated plate is inserted in the brain tissue to measure blood flow via thermal conduction [16]. Limited articles have been published concerning TDF and the utility is stated to be limited as TDF is an invasive modality with a small field of examination and uncertainly concerning probe placement [30].

Radiological estimation of CBF via xenon CT, PET, perfusion-CT, or -MRI can only provide snapshots of CBF. Disadvantages depend on the specific modality and include radiation exposure (CT), risk of allergic reaction to contrast, renal affection (perfusion scans), low availability, and high costs (PET and MRI) [14, 44]. Both methods were uncommon in the Nordic countries.

### The extent of use and level of agreement

Analyzing in-center agreement, the consensus is low concerning clinical routines and research methods between the appointed experts’ benchmark and their respective trainees. This may reflect low prioritization of training regarding neurointensive care monitoring, although in-center disagreement regarding department treatment policies has also been observed in other aspects of patient care in Neurosurgical departments [6]. Missing answers among trainees were highest when asking about the attitudes and main issues regarding aMMM (9% and 7%) which might indicate ambivalence to having an opinion when reflecting on this complex issue, instead of lacking knowledge. Regarding the use of modalities, missing answers were highest when asking about NIRS, and TDF (6% and 4%), the two least used methods, which could indicate a lack of experience.

As robust evidence regarding outcome-changing effects for most monitoring methods is lacking, the use of monitoring depends on center preferences, economy, infrastructure, and research activity [32]. This fact is reflected in the inter-center disagreement level. Only around 50% of respondents implied PbtO_2_ and/or CMD to be a sound clinical practice allowing early detection of secondary brain injury in severe TBI or SAH. To some degree, academic disagreement is a factor in the implementation of aMMM, besides financial factors.

The use of aMMM has also been investigated in a European survey from 2017 including 66 neurotrauma centers. All modalities were used less compared to the Nordic countries; TCD 75% vs 38%, PbtO_2_ 53% vs 19%, SjvO_2_ 19% vs 9%, CMD 12% vs 6%, and NIRS 12% vs 2% [9]. This may be due to a general development over time or a higher use in the Nordic countries. However, SAH patients were not included in the study, which could explain the lower use of TCD.

### Knowledge, opinions, and experiences regarding the use of aMMM

Most respondents claimed knowledge of CMD mechanisms but only a few were confident in the interpretation and use of PbtO_2_ and CMD, indicating a lack of knowledge, interest, or clear guidelines (Table 2).

#### Main concerns according to the respondents

11% of respondents rated risk-related issues as the main concern. However, the risk of implanting PbtO_2_ has been investigated in a systematic review from 2014, which found no catheter-related infections, only 0–3% local bleeding (with no clinical consequence), and 6–14% technical complications [37].

The main issue with aMMM was stated to be usefulness regarding outcome-changing interventions by 43% of respondents. Insufficient evidence has resulted in few guideline recommendations. Although consensus statements and algorithm-guided therapy could be a way to facilitate implementation, it can also be argued that the better understanding of pathophysiological mechanisms learned from neuromonitoring is the fundament of modern neurocritical care which has already reduced mortality dramatically [35] and is quite possibly still the best way to advance treatments in the future.

#### Future considerations

Most respondents (67%) believed automated combined analysis of aMMM to be a likely development and 19% had first-hand experience with implementing machine-learning algorithms and/or AI in a neuromonitoring context.

Threshold-based alarms from single monitors often provide insufficient information [45]. Implementing AI or automated continuous measurements could increase the performance and accuracy of the modalities [12, 39]. Furthermore, it could help integrate data from various measurements, thereby helping predict events, discover trends, and facilitate utilization in the intensive care unit [26]. This would especially be useful in SD and DCI detection using cEEG, which require time and expertise to interpret. However further studies examining the performance of scalp EEG compared to cortical electrodes are needed [7].

### Limitations

Sources of bias in this questionnaire survey include recall bias, and misinterpretation of questions due to formulation errors or respondents’ perception.

Despite the perfect response rate, a trainee coming into the field of aMMM in neurointensive care may find it difficult to assess which modalities are considered standard of care at their home center and generalizability may be low. Therefore, appointed experts in neurointensive care monitoring from each neurosurgical center were included under the assumption that their answers reflect the most objective estimation.

## Conclusions

Although certain additional neuromonitoring modalities are favored by most neurosurgical centers in the Nordic countries, there are great variations in the extent of use and a high level of in-center disagreement between experts and trainees. In-center disagreement among trainees may reflect low prioritization of aMMM in Nordic neurosurgical training, which in turn may affect future development in the field. Several known and market-approved monitoring modalities seem to be scarce in use, and neurosurgical trainees and experts question their value.

This study provides insight regarding neuromonitoring prioritization in the Nordic countries, sheds light on perceived obstacles, and may aid in inter- and in-center cooperation regarding an important cornerstone for the development of modern neurointensive care.

## Data Availability

Data is provided within the manuscript or supplementary information files.

## Abbreviations

BTF: Brain Trauma Foundation
CBF: Cerebral blood flow
cEEG: Continuous electroencephalography
CMD: Cerebral microdialysis
ICP: Intracranial pressure
LPR: Lactate to pyruvate ratio
MMM: Multimodal neuromonitoring
NIRS: Near-infrared
PbtO_2_: Brain tissue oxygenation
SD: Spreading depolarization
SjvO_2_: Jugular bulb oxygen saturation
TCD: Transcranial Doppler ultrasound
TDF: Thermal diffusion flowmetry
